# Models and Approaches for Comprehension of Dysarthric Speech Using Natural Language Processing: Systematic Review

**DOI:** 10.2196/44489

**Published:** 2023-10-27

**Authors:** Benard Alaka, Bernard Shibwabo

**Affiliations:** 1 School of Computing and Engineering Sciences Strathmore University Nairobi Kenya

**Keywords:** dysarthria, speech comprehension, speech contextualization, meaning extraction, ontology extraction, familiarity, topic knowledge

## Abstract

**Background:**

Speech intelligibility and speech comprehension for dysarthric speech has attracted much attention recently. Dysarthria is characterized by irregularities in the speed, strength, pitch, breath control, range, steadiness, and accuracy of muscle movements required for articulatory aspects of speech production.

**Objective:**

This study examined the contributions made by other studies involved in dysarthric speech comprehension. We focused on the modes of meaning extraction used in generalizing speaker-listener underpinnings in light of semantic ontology extraction as a desired technique, applied method types, speech representations used, and databases sourced from.

**Methods:**

This study involved a systematic literature review using 7 electronic databases: Cochrane Database of Systematic Reviews, Web of Science Core Collection, Scopus, PubMed, ACM, IEEE Xplore, and Google Scholar. The main eligibility criterion was the extraction of meaning from dysarthric speech using natural language processing or understanding approaches to improve on dysarthric speech comprehension. In total, out of 834 search results, 30 studies that matched the eligibility requirements were acquired following screening by 2 independent reviewers, with a lack of consensus being resolved through joint discussion or consultation with a third party. In order to evaluate the studies’ methodological quality, the risk of bias assessment was based on the Cochrane risk-of-bias tool version 2 (RoB2) with 23 of the studies (77%) registering low risk of bias and 7 studies (33%) raising some concern over the risk of bias. The overall quality assessment of the study was done using TRIPOD (Transparent Reporting of a Multivariable Prediction Model for Individual Prognosis or Diagnosis).

**Results:**

Following a review of 30 primary studies, this study revealed that the reviewed studies focused on natural language understanding or clinical approaches, with an increase in proposed solutions from 2020 onwards. Most studies relied on speaker-dependent speech features, while others used speech patterns, semantic knowledge, or hybrid approaches. The prevalent use of vector representation aligned with natural language understanding models, while Mel-frequency cepstral coefficient representation and no representation approaches were applied in neural networks. Hybrid representation studies aimed to reconstruct dysarthric speech or improve comprehension. Comprehensive databases, like TORGO and UA-Speech, were commonly used in combination with other curated databases, while primary data was preferred for specific or unique research objectives.

**Conclusions:**

We found significant gaps in dysarthric speech comprehension characterized by the lack of inclusion of important listener or speech-independent features in the speech representations, mode of extraction, and data sources used. Further research is therefore proposed regarding the formulation of models that accommodate listener and speech-independent features through semantic ontologies that will be useful in the inclusion of key features of listener and speech-independent features for meaning extraction of dysarthric speech.

## Introduction

The comprehension of dysarthric speech in usual discourse goes beyond basic recognition of the words uttered by the speaker. Effective comprehension acknowledges the legacy communication structure of speaker-channel-listener, with a focus on the intended message. As such, it is important to figure out the intended message of the dysarthric speaker, particularly after the recognition of the words uttered. The nature of dysarthric speech makes the comprehension task graver following the adaptive means taken up by such speakers, which may only be understood better by listeners who are familiar with their manner of expression and choice of vocabulary [1,2]. Having knowledge of the topic of discussion during discourse may be helpful in understanding what the patient with dysarthria is saying; however, it has been shown that even the patterns of intertopic switching by these speakers are outside of the usual discourse norms. As such, the primary task of listener-targeted remediation in offsetting the intelligibility burden associated with dysarthria from the speaker is left with the listener [3,4].

Speech contextualization is the most direct approach taken toward comprehending speech. Linguistically, context refers to a part of the real world where certain events or conversations occur, and it is frequently mistaken with another meaning, namely, knowledge about the same thing [5]. To discern context, there must be some common information between the speaker and the listener, at least to an acceptable degree. The concepts of comprehensibility and intelligibility may be distinguished by the fact that comprehensibility incorporates signal-independent information, such as syntax, semantics, and physical context. This distinction implies that the ability of a listener to retrieve the semantic code of spoken speech is dependent on both the acoustic-phonetic information and all relevant signal-independent information [6]. This dependence influences the main assumption of this study that events occurring within and without the speech itself are crucial for deciphering the intended meaning of the words spoken, thereby informing the context of the situation.

With the recent growing interest in the explicit modelling of events in structural conceptual models, ontology extraction has become one of the formidable trends and tools of use for explicitly representing events in structural models [7]. Ontologies are mainly perceived as a knowledge graph able to formally model different aspects of our real world [8]. A common issue with these ontologies is that, being manually crafted and maintained by domain experts, they tend to evolve relatively slow and become quickly outdated. To keep up with the pace of the constant evolution of the research landscape, some institutions are crowd-sourcing their classification scheme [9]. More efficient techniques, such as the use of self-learning vector machines, have been proposed to counter these shortcomings [10].

Speech comprehension as implemented in neural machine translation and automated speech recognition models are mostly ineffective as attributed by their universal assumptions that tend to factor out the listener or intended listener while trying to locate context from isolated sentences or in some cases interrelated sentences from the same speaker [11,12]. The close standing techniques, such as semantic projection, assume similarity in vocabulary level between the speaker and listener with little emphasis put on levels of familiarity or topic sentence as the bridge between the message source and the intended recipient [1,2].

The fundamental structure for these solutions, which is word vector mapping, bears few pointers that could aid word embedding in informing meaning when plugged into any sort of machine translator, whether affective or attention-based [5,13]. These challenges are more elaborate in dysarthric speech where speech listeners are more likely to create linguistic generative models for new talkers based on their understanding of the distribution of auditory cues associated with each linguistic category [14].

Therefore, this study reviewed models and methodologies that are used in comprehending meaning from audio dysarthric speech by means of speech-event representations, otherwise known as situational projections. Following the fact that familiarization formed the core paradigm of the reviewed works as a technique that affords listeners an opportunity to retune their stored linguistic representations [15,16], the following key research question arose: following the unique characteristics of each speaker, how do the reviewed models generalize over new dysarthric speech given the specificity of the speaker? This study discusses the approaches used in generalizing speaker-listener underpinnings in relation to familiarization in light of ontology extraction as a technique of interest.

## Methods

### Goal and Review Questions

#### Goal of the Study

The goal of this study was to systematically map (classify), review, and synthesize studies that focus on the use of natural language processing and natural language understanding (NLU) in extracting meaning from dysarthric speech. Moreover, this study aimed to detect recent trends and directions of the field to identify opportunities for future research from both researchers’ and practitioners’ perspectives. Guided by the established guidelines for conducting systematic literature review studies [17] and the established procedure for reporting the findings of the review [18,19], the selection and review process was developed and reported. Based on the above goal, review questions (RQs) were raised and grouped under 1 of 2 categories.

#### Category 1 RQs

The following RQs were general to all systematic literature review studies:

1. RQ 1.1. Mapping of studies by contribution types: what are the contributions presented in the studies? The motivation for this RQ is that the contribution identified in the respective studies largely influences the nature of the proposed solution regarding whether they are approaches, methods, or tools for contextualizing or comprehending dysarthric speech.
2. RQ 1.2. Mapping of studies by research method types: how many studies presented empirical or theoretical frameworks? Following the multidisciplinary nature of this study, it was likely that the manner of comprehending dysarthric speech would vary, hence this RQ.

#### Category 2 RQs

The following RQs were specific to the topic (contextualizing dysarthric speech):

1. RQ 2.1. The mode of meaning extraction used: what modes of meaning extraction were used in the models and approaches of the reviewed studies? The main rationale for this RQ was to investigate how different studies tended to include or exclude the listener decoding process from the meaning extraction process as influenced by both their inputs and output.
2. RQ 2.2. The nature of word representations used: what is the nature and ability of the word representation used in the meaning extraction from dysarthric speech? Due to the ability of different word representations used within the reviewed studies, such as word vectors, context vectors, and ontology web vectors, it was necessary to ask this question.
3. RQ 2.3. Data sources: what data sources were used to train the meaning extraction models? This RQ was formulated for the purpose of assessing the richness of the solution offered by the reviewed studies.

### Search Strategy

This systematic review was reported in accordance with the PRISMA (Preferred Reporting Items for Systematic Reviews and Meta-Analyses) guidelines [18,19]. A comprehensive literature search was conducted on 7 databases from inception to 2023 to identify relevant articles. The databases searched included the Cochrane Database of Systematic Reviews, Web of Science Core Collection, Scopus, PubMed, ACM, IEEE Xplore, and Google Scholar for studies reporting meaning extraction tools, approaches, and methods for dysarthric speech.

**Figure 1:**
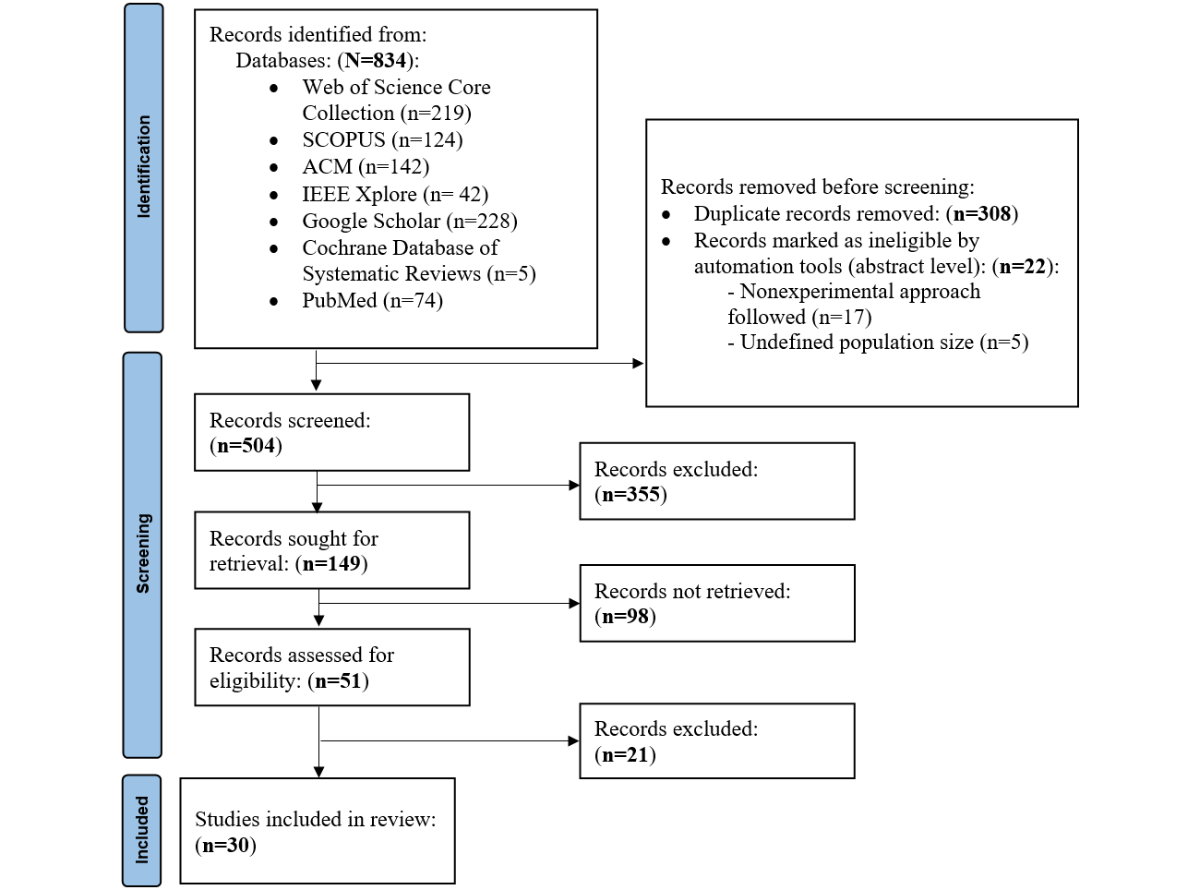
PRISMA (Preferred Reporting Items for Systematic Reviews and Meta-Analyses) flowchart for the selection of systematic review literature.

The selection of articles is presented in Figure 1. Further, we defined search strings for each database searched, in accordance with the PICO (Problem, Intervention, Comparison, and Outcome) structure [20] (Multimedia Appendix 1). The keywords for the PICO structure were guided by the general question “can listener-based natural language processing models and approaches improve the comprehension of dysarthric speech?”

The following sentences further describe the PICO structure followed:

1. Problem: studies related to dysarthria or dysarthric speech and variations (“dysarthria” OR “dysarthric” OR “dysarthrias” OR “dysarthrics”).
2. Intervention: studies related to natural language processing approaches or techniques used for speech comprehension and their synonyms or abbreviations (“natural language processing” OR “NLP” OR “natural language understanding” OR “NLU” OR “automated speech recognition” OR “intelligibility” OR “listener*” OR “listen” OR “technique” OR “approach”).
3. Comparison: given the significant difference in our objectives and goals from those of past research on the topic, there were no comparable studies to use as a baseline.
4. Output: comprehension tasks and synonyms arising from the use of natural language processing approaches (“comprehension” OR “meaning” OR “contextualization*” OR “context*” OR “comprehension*” OR “comprehend” OR “understand”).

Using “AND” and “OR” operators to link these ideas, we came up with the following general search phrase: (“Dysarthria” OR “Dysarthric” OR “Dysarthrias” OR “Dysarthrics”) AND (“natural language processing” OR “NLP” OR “natural language understanding” OR “NLU” OR “automated speech recognition” OR “intelligibility” OR “listener*” OR “listen” OR “technique” OR “approach”) AND (“comprehension” OR “meaning” OR “context” OR “contextualization” OR “comprehend” OR “understand”). Slight variations depending on the databases searched are documented in Multimedia Appendix 2.

### Inclusion and Exclusion Criteria

Out of 834 search results, a total of 30 studies were included for review. Studies that were included must have fulfilled the following criteria: (1) studies that focused on meaning extraction and not just on intelligibility, (2) studies that focused on dysarthric speech, and (3) studies that clearly defined approaches, tools, or methods for dysarthric speech comprehension.

Studies that were excluded included the following: (1) literature investigating other speech disorders, such as aphasia; (2) studies which conducted other speech tasks, such as measuring the severity of dysarthria, classification of dysarthric type, and assessment of the speaker’s dysarthric level; (3) literature that did not apply natural language processing interventions, such as speech therapy rehabilitation or clinical approaches; (4) literature that focused on dysarthric speech features or characteristics, leaving out dysarthric speech patterns; and (5) studies that solely focused on dysarthric speech intelligibility and not its comprehensibility.

CADIMA systematic review software [21] was used for screening automation. A 2-stage screening process was used. To reduce bias, 2 reviewers screened all titles and abstracts. The first step assessing relevance to the inclusion criteria was performed by the 2 researchers independently, and the papers passed to the next step if at least 1 reviewer decided so. In the events where consensus at the title and abstract level screening could not be achieved between the 2 reviewers an adjudication sourced from a third person on a consultation basis was sought.

During the second stage, 2 independent reviewers performed a full-text blind review, with consensus obtained after deliberation between reviewers, when required. The consensus meetings were held to resolve disagreements and uncertainty. Finally, the objectivity of the criteria was assessed, either prereview on a test set by measuring agreement or postreview.

### Data Synthesis and Analysis

As per the RQs, the following information points were recorded and analyzed: (1) year of publication, (2) contribution of each study, (3) categorization of each study per the relevant research method type, (4) mode of meaning extraction used in each study, (5) nature of word representation used in light of their abilities to effectively extract meaning from dysarthric speech, (6) speech patterns considered, and (7) data sources used in the studies. Tables and graphs were developed to summarize the information points.

### Quality Assessment

Quality assessment was conducted by 2 independent reviewers on 2 levels. First, the overall assessment of the quality of studies was chosen to be reviewed, and second, the risk of bias (ROB) was assessed. After completing the independent assessments, areas of discrepancies in each of the reviewers’ evaluations were identified. A constructive discussion of the identified discrepancies ensued for purposes of highlighting the reasons for the differing assessments. Active reference to the study protocol used [22] formed the main basis for a consensus to be achieved during these discussions.

The goal of the quality assessment was to determine the significance of each chosen document. We described the evaluation largely to reflect the validity of the chosen studies, even if the quality rating had no bearing on the choice of the primary investigations. The study’s compliance with the TRIPOD (Transparent Reporting of a Multivariable Prediction Model for Individual Prognosis or Diagnosis) [22] standard served as a gauge of its quality. Each article was given 1 or 0 points depending on how closely the TRIPOD checklist was followed or not. Additionally, every paper that was reviewed scored higher than 50%. For more information on the outcomes of the TRIPOD assessment standards, see Multimedia Appendix 3.

**Figure 2:**
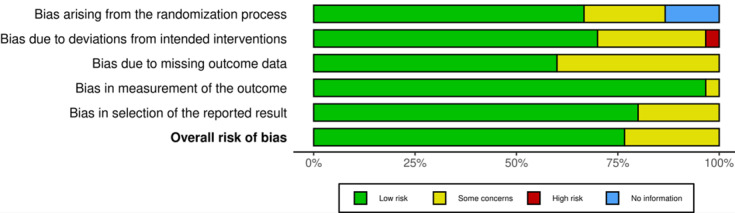
Summary of the risk of bias assessment.

Given that the participants of the studies reviewed patients with dysarthria, the Cochrane RoB2 tool was used to assess the ROB. Results of the domain-specific and overall ROB assessment ratings of the set of 30 studies are shown in Figure 2. The individual ROB ratings of all studies are included in Multimedia Appendix 4. The ROB was assessed using 5 domains, including randomization process bias, deviation from intended outcome bias, missing outcome data bias, measure of outcome bias, and selection of reported results bias.

**Figure 3:**
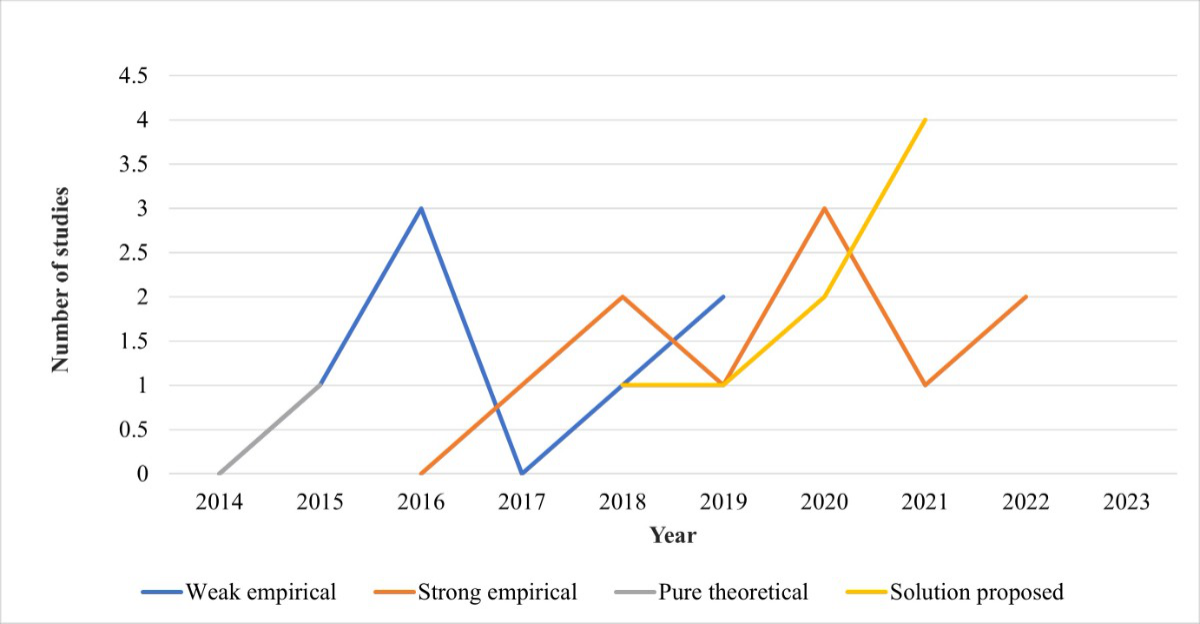
Cumulative trend of the mapping of studies by research method type.

In the randomization process bias domain, 20 (67%) studies were rated as low ROB, 6 (20%) as causing some concern, and 4 (13%) as bearing no information needed. These ratings indicated that little bias occurs when a trial’s results are affected by human choices or other factors not related to the treatment being tested. In the deviation from intended outcome bias domain, 21 (70%) studies were rated as low ROB due to a majority of the studies being specific in their outcome either as actual solutions or empirical solutions, weak or strong as they may be (Figure 3). Furthermore, 8 (27%) studies were rated as causing some concern and only 1 (3%) study as bearing high ROB. This may be attributed to the primary goal of this review where the studies selected were focused on achieving comprehensibility, yet a majority tended to skew more toward intelligibility.

The missing outcome data bias domain comprised the lowest proportion (n=18; 60%) of low ROB ratings among all 5 domains in our investigation and the highest proportion (n=12; 40%) of somewhat concerning ROB ratings. This may have been posed by a majority of studies not reporting the clear state or data type of the data churned out of their proposed solution for use in dysarthric speech comprehension. The measure of outcome bias domain comprised the highest proportion (n=29; 97%) of low ROB ratings among all 5 domains in our investigation. Only 1 (3%) study somehow concerns ROB. The main reasons for the high ROB were strong internal and external validation used in a majority of the studies as a means of quantifying the effectiveness of the proposed solutions therein.

In the selection of reported results bias, 24 (80%) studies had low ROB with 6 (20%) of the studies causing some concern over the ROB. Overall, only 23 (77%) studies received a low ROB rating, whereas 7 (33%) studies were judged to have a somewhat concerning ROB. Of these 7 studies, 1 [23] received a high ROB rating on deviation from intended outcome as intelligibility rose as its core objective instead of comprehensibility.

## Results

This section is structured according to the classification of studies and technical issues regarding the contextualization of dysarthric speech.

### Classification of Studies

First, the studies were classified according to their contribution. We discovered 4 apparent contributions from the studies: (1) hybrid (a combination of clinical and NLU approaches), which formed approximate 17% (n=5) of the pool; (2) NLU techniques, which formed 43% (n=13) of the pool; (3) theoretical approaches, which formed 10% (n=3) of the pool; and (4) clinical therapy approached, which formed 30% (n=9) of the pool. Generic acoustic tools and theoretical tools were grouped into 1 category following the fact that the acoustic concepts applied in both studies were similar.

The studies were mapped by research method type. The identified research methods from the studies were weak empirical studies, strong empirical studies, pure theoretical studies, and studies bearing proposed solutions that intended to derive meaning from dysarthric speech. As illustrated in the cumulative trend diagram in Figure 3, earlier studies (2015-2019) attracted a majority of weak empirical studies on dysarthric speech comprehension, with strong empirical studies having their onset from 2016 onwards. The distribution of pure theoretical studies was sparse through the years, while studies proposing actual solutions for meaning extraction from dysarthric speech appeared mainly from 2018 onwards.

### Technical Issues Specific to the Topic (Contextualizing Dysarthric Speech)

#### Mode of Meaning Extraction Used

It was important to establish the mode of meaning extraction from dysarthric speech to aid in showing how different studies acknowledged the role of the listener in remediating dysarthric speech. The mode of meaning included the specific speech inputs into the proposed models. Studies that solely used speech features [15,24-35] were heavily speaker-dependent on their approach and tended to lean more toward intelligibility of the dysarthric speaker than their comprehensibility.

Studies that emphasized speech patterns [23,27,36-42] were interested in the formation of words spoken, omissions in their patterns, and inclusion of interesting vocabulary during discourse. These studies were much aligned toward word representation and drawing meaning out of the same by leveraging other speech-independent features, such as the speakers’ emotions. Closely tied to studies that relied on speech pattern were studies that used semantic knowledge [43,44] that mostly worked by combining language rules, background knowledge, and semantic change patterns so as to understand semantic similarity-based relevance between questions and corresponding answer sentences.

Finally, studies that had a hybrid approach [23,45-51] combined speech features, speech patterns, and semantic knowledge so as to go beyond the mere task of intelligibility and pose questions on possible comprehensibility of the speech being studied. These studies incorporated the use of familiarization and topic knowledge techniques.

#### Nature of Speech Representations Used

Investigating the nature of the word representation was instrumental in establishing the robustness of the approaches, tools, or solutions proposed in the studies reviewed. Whereas some representations, such as Fourier transformation and Mel-frequency cepstral coefficients (MFCC), are popular in speech processing, their abilities to handle dimensions of semantic knowledge are questionable [5,13]. It is worth noting that for most studies that used MFCC [28,29,31,35,36,47], variations of artificial neural networks were used in all except one [26], in which a stochastic model was applied.

This uniformity was unlike the case in studies that used no representation at all [24,25,27,30,32,37,44,46] in which a mix of models ranging from hybrid (neural models and clinical approaches) to support vectors were used. The tasks of this category mostly involved assessment of intelligibility in domain specific cases of dysarthria, such as Parkinson disease, or any other noise, with comprehensibility being a secondary or aiding factor.

The studies that used vector encoding [15,38-43,50,51] used NLU-based models, such long short-term memory neural networks or combinations of gated recurrent unit and convolutional neural networks to achieve the tasks of dialogue assessment in dysarthric speech, language understanding, and semantic pattern tracking.

Finally, studies that used a hybrid approach [23,33,34,45,48,49] combined MFCC with variations of vector encoding. These studies were characterized by variations of models, such as adversarial networks, support vector machines, gated recurrent unit and convolutional neural networks, and hidden Markov models. The tasks in each of these studies also varied and were very heavily geared toward reconstruction of dysarthria through assessment of the semantics presented.

#### Databases Used

The choice of the database used was assessed to ascertain the depth of the approach, tool, or solution proposed in the reviewed studies. This was important in informing this study of the flexibility of data used in achieving dysarthric speech comprehensibility. Extensive and well-documented databases, such as the TORGO database and the UA-Speech database, contain data sets that have the potential to yield the in-depth speech patterns necessary for speech comprehension. Studies that used these 2 databases [26,31] (n=2; 6% of the studies) or a hybrid of any other databases (n=11; 37% of the studies) [28,33,37-41,47-49,51] focused more on the application of the data in their proposed models, with little effort going into curation and preprocessing of the data.

However, a majority of the reviewed studies (n=17; 57% of the studies) involved audio data that were primarily sourced. These studies [15,23-25,27,29,30,32,34-36,42-46,50] foremost curated the data and augmented their input data representations to accommodate features that ordinary speech representations would not have accommodated in their natural form. Further models for speech processing were developed and these representations were used to train the models.

## Discussion

### Summary of Findings

The ultimate purpose of this study was to gain an in-depth understanding of the current state of research in remote sensing for the comprehension of dysarthric speech, to give suggestions about future lines of research, and to find new possibilities and application areas. This can be achieved by an analysis and discussion of the results presented in the previous section.

The mapping of contributions made by the reviewed studies indicated that mostly NLU or clinical approaches were used. This was evidenced in the findings of reviewed method types where the number of actual proposed solutions only began to rise in 2020, having been preceded by empirical studies that perhaps meant to justify the suitability of the clinical approaches used.

The mapping of studies by method types also indicated an increase in theoretical research from 2020 onwards, which bore the theoretical framework necessary for justifying the new actual proposed solutions for extracting meaning from dysarthric speech. Studies preceding 2018 were mostly characterized by weak empirical or pure theoretical studies.

It is worth noting that a majority of the studies heavily relied on speaker-dependent speech features, thereby leaning more toward intelligibility with little focus on the semantics of the speech input. Studies that solely used speech features were biased toward performing dysarthric speech intelligibility tasks as opposed to comprehensibility. This was contrary to studies that used speech patterns, semantic knowledge, or a hybrid of the two, which aimed at applying rules, background knowledge, and familiarity features in order to comprehend dysarthric speech.

The use of vector representation was a prominent speech representation used in a majority of the studies; this was in tandem with the findings of the contributions made by the reviewed studies, given that most natural language processing models or approaches preferred to use vectors and no representation approach solutions. Whereas vector-based representations were largely applied in NLU models, MFCC representations as well as studies that applied no representation were mostly applied in variations of artificial neural networks or a mix thereof. The studies that applied hybrid representation were unique in the sense that they delved more toward reconstructing dysarthric speech or assessing approaches for reconstructing dysarthric speech so as to achieve better meaning.

Comprehensive databases, such as TORGO and UA-Speech, were rarely used in solitude; their combination with other curated databases resulted in a slightly higher number of hybrid data sources compared to TORGO or UA-Speech independently. The majority of studies, however, used primary data curated for specific tasks intended for the objective.

### Implications

With NLU studies gaining traction from 2020 onwards, and a number of NLU approaches being hybridized with clinical approaches, there is an inference that dysarthria is indeed deemed a major medical condition warranting a similar approach [52-54]. This is pegged heavily on the need of such approaches to be dependent on human intervention, which has been the norm over the past few years of dysarthric speech research.

This posits that the proposition of a speech comprehensibility solution for dysarthric speech may, both in the present and future, rely on clinical findings to inform the models being proposed. While this dependence on clinical approaches may possibly be seen as being problematic to potential stand-alone NLU approaches, it may be appraised as a potential strength for informing the overall generalizability of the approaches, particularly with regard to providing speaker-specific meaning extraction [12,55]. Where generalizability is high, the human intensive effort that would have been put in becomes rigorous. As such, there arises a need for models that are both speech-dependent and speech-independent to allow for a variation of features necessary for achieving comprehension [2].

There has been limited breakthrough in the study of speech comprehension models with the onset of actual proposed solutions from 2020 onwards, as the findings indicate that a merely theoretical approach was followed, meaning that the output of these studies are theoretical frameworks as opposed to actual solutions. This perhaps may be tied to either a lack of sufficient data needed as primary inputs to the proposed frameworks or a narrow technical gap presented by the same [26,56]. This can be reflected by the vast use of primary data by most of the reviewed studies as opposed to existing databases, perhaps out of a lack of sufficient data within the existing dysarthria speech databases.

The slim gap problem faced by existing theoretical frameworks involves the significant assumption made that intelligibility is similar to comprehensibility [57]. This is reflected by much reliance on speech features as opposed to speech patterns that would require more data points to draw meaning. This assumption has resulted in the formulation of representations that are not sufficient to perform a speech contextualizing task, which goes beyond a simple translation of the words spoken [5,13]. It is suggested that intelligibility be treated as an objectively different task from comprehensibility, which in this study is deemed as the message derived from speech given both speech-dependent and speech-independent factors.

Additionally, the limitation in the gap for the presently existing theoretical frameworks may first be attributed to a biased definition of redefining familiarity, which alludes to a heavy connotation in communication theory. There is need to redefine familiarity as a projection of word feature vectors into the d-dimensional semantic word space with consideration of a set of classes that constitute several situational markers, such as topic events and emotional events [7,58]. This shall be useful to natural language models that seek to incorporate such vectors as inputs necessary for inferring context of speech and thereby comprehending speech.

### Limitations

This study is limited to only a review of 30 studies by virtue of the scope of the study that sought to review methods and approaches used to comprehend dysarthric speech using techniques of natural language processing. The strict definition of comprehension left out a number of studies whose task solely focused on intelligibility as a task. Additionally, the study was limited to the previously discussed research questions and as such did not address other prominent techniques, such as the use of additional techniques (eg, computer vision) while attempting to comprehend dysarthric speech. This followed the fact that the comprehension of speech could be limited to a few speech-related events for the purposes of meaning extraction, as much as additional nonspeech features would enrich the discussed methods and approaches.

### Conclusion

By reviewing the relevant studies, this systematic review mapped and reviewed the body of knowledge on studies that attempted to extract meaning from the inputs and generic nature of dysarthric speech. In total, 30 papers were systematically reviewed and synthesized in accordance with the formulated RQs. Following this summary, this paper provides an index of the vast body of knowledge in this area. An important finding ensuing from this study was that actual meaning extraction was minimal, with a majority of the studies leaning toward speech intelligibility solely. This general finding is important as it informs communication scholars and dysarthria clinical experts of the crucial need to include the listener as a party during meaning extraction experiments. This finding is also important for NLU experts who need to formulate representations that are robust enough to incorporate listener factors, such as familiarity, topic knowledge, and nonspeech events, that may bear pointers toward the meaning of dysarthric speech. Therefore, this study presents to the science community the need for further research with regard to the formulation of semantic ontologies that will be useful in NLU for meaning extraction.

## Appendix

Multimedia Appendix 1 PICO (Problem, Intervention, Comparison, Outcome) framework of the research questions.

Multimedia Appendix 2 Databases and search terms used in the literature search.

Multimedia Appendix 3 TRIPOD (Transparent Reporting of a Multivariable Prediction Model for Individual Prognosis or Diagnosis) checklist and results for quality assessment of the prediction models.

Multimedia Appendix 4 Cochrane risk of bias. Assessment of the risk of bias for each study.

## Abbreviations

MFCC: Mel-frequency cepstral coefficient
NLU: natural language understanding
PICO: Problem, Intervention, Comparison, and Outcome
PRISMA: Preferred Reporting Items for Systematic Reviews and Meta-Analyses
ROB: risk of bias
RQ: review question
TRIPOD: Transparent Reporting of a Multivariable Prediction Model for Individual Prognosis or Diagnosis
