# Supplementary material for: Models and Approaches for Comprehension of Dysarthric Speech Using Natural Language Processing: Systematic Review
Source: JMIR Rehabil Assist Technol. 2023 Oct 27;10:e44489. doi: 10.2196/44489 (PMC10655903; doi:10.2196/44489)
Supplement: Multimedia Appendix 1 [file rehab_v10i1e44489_app1.docx]

**Multimedia Appendix 1: Problem, Intervention, Comparison, Outcome (PICO) Framework of Research Questions**

| PICO | Concepts | Related terms for search |
| --- | --- | --- |
| Problem | Dysarthria | Dysarthria, Dysarthric Speech, Dysarthric, Dysarthrics, Dysarthrias  Clinical research, clinical research protocol, clinical studies |
| Intervention | Natural Language Processing Techniques/Approach | Natural language processing (NLP), Natural Language Understanding (NLU), Automated Speech Recognition (ASR), Speech Comprehension, Speech Intelligibility, Meaning Extraction, Techniques, Approach, Model |
| Comparison | N/A | N/A |
| Outcome | Improved Comprehension | Contextualization, Meaning, Comprehension |

*Note*. To enable a comprehensive search and avoid excluding relevant studies and given that there was no alternative intervention other than Natural Language Processing, the comparison was not included in the search terms.
