# Supplementary material for: Models and Approaches for Comprehension of Dysarthric Speech Using Natural Language Processing: Systematic Review
Source: JMIR Rehabil Assist Technol. 2023 Oct 27;10:e44489. doi: 10.2196/44489 (PMC10655903; doi:10.2196/44489)
Supplement: Multimedia Appendix 2 [file rehab_v10i1e44489_app2.docx]

**Multimedia Appendix 2: Databases and Search Terms Utilized in Literature Search**

| Database | Search terms | Publication retrieved |
| --- | --- | --- |
| PubMed | (“Dysarthria” [MESH] OR “Dysarthric” OR “Dysarthrias” OR “Dysarthrics”) AND (“natural language processing” OR “NLP” OR “natural language understanding” OR “NLU” OR “automated speech recognition” OR “intelligibility” OR “listener*” OR “listen” OR “technique” OR “approach”) AND (“comprehension” OR “meaning” OR “context” OR “contextualization” OR “comprehend” OR “understand”) | 74 |
| Cochrane Database of Systematic Reviews (CDSR) | (“Dysarthria” OR “Dysarthric” OR “Dysarthrias” OR “Dysarthrics”) AND (“natural language processing” OR “NLP” OR “natural language understanding” OR “NLU” OR “automated speech recognition” OR “intelligibility” OR “listener*” OR “listen” OR “technique” OR “approach”) AND (“comprehension” OR “meaning” OR “context” OR “contextualization” OR “comprehend” OR “understand”) | 5 |
| Google Scholar | (“Dysarthria” OR “Dysarthric” OR “Dysarthrias” OR “Dysarthrics”) AND (“natural language processing” OR “NLP” OR “natural language understanding” OR “NLU” OR “automated speech recognition” OR “intelligibility” OR “listener*” OR “listen” OR “technique” OR “approach”) AND (“comprehension” OR “meaning” OR “context” OR “contextualization” OR “comprehend” OR “understand”) | 228 |
| IEEE Xplore | (“Dysarthria” OR “Dysarthric” OR “Dysarthrias” OR “Dysarthrics”) AND (“natural language processing” OR “NLP” OR “natural language understanding” OR “NLU” OR “automated speech recognition” OR “intelligibility” OR “listener*” OR “listen” OR “technique” OR “approach”) AND (“comprehension” OR “meaning” OR “context” OR “contextualization” OR “comprehend” OR “understand”) | 42 |
| ACM | [[All: allfield] OR [All: "dysarthria"] OR [All: "dysarthric"] OR [All: "dysarthrias"] OR [All: "dysarthrics"]] AND [[All: allfield] OR [All: "natural language processing"] OR [All: "nlp"] OR [All: "natural language understanding"] OR [All: "nlu"] OR [All: "automated speech recognition"] OR [All: "intelligibility"] OR [All: "listener*"] OR [All: "listen"] OR [All: "technique"] OR [All: "approach"]] AND [[All: allfield] OR [All: "comprehension"] OR [All: "meaning"] OR [All: "context"] OR [All: "contextualization"] OR [All: "comprehend"] OR [All: "understand"]] | 142 |
| SCOPUS | (“Dysarthria” OR “Dysarthric” OR “Dysarthrias” OR “Dysarthrics”) AND (“natural language processing” OR “NLP” OR “natural language understanding” OR “NLU” OR “automated speech recognition” OR “intelligibility” OR “listener*” OR “listen” OR “technique” OR “approach”) AND (“comprehension” OR “meaning” OR “context” OR “contextualization” OR “comprehend” OR “understand”) | 124 |
| Web of Science Core Collection | (“Dysarthria” OR “Dysarthric” OR “Dysarthrias” OR “Dysarthrics”) AND (“natural language processing” OR “NLP” OR “natural language understanding” OR “NLU” OR “automated speech recognition” OR “intelligibility” OR “listener*” OR “listen” OR “technique” OR “approach”) AND (“comprehension” OR “meaning” OR “context” OR “contextualization” OR “comprehend” OR “understand”) | 219 |
| **TOTAL** |  | **834** |
