# Supplementary material for: Models and Approaches for Comprehension of Dysarthric Speech Using Natural Language Processing: Systematic Review
Source: JMIR Rehabil Assist Technol. 2023 Oct 27;10:e44489. doi: 10.2196/44489 (PMC10655903; doi:10.2196/44489)

**Multimedia Appendix 4a: Assessment of Risk Bias for Each Study**

| **Author, Year [REF]** | **Randomization Process** | **Deviation from Intended Outcome** | **Missing Outcome Data** | **Measurement of Outcome** | **Selection of Reported Results** | **Overall** |
| --- | --- | --- | --- | --- | --- | --- |
| Alhiniti et al., 2021 [24] | Low | Low | Low | Low | Low | Low |
| Alhiniti et al., 2020 [36] | Some concerns | Low | Low | Low | Low | Low |
| Allison & Hustad 2018 [25] | No information | Low | Some concerns | Low | Low | Low |
| Cardoso et al., 2017 [43] | Low | Low | Some concerns | Low | Some concerns | Some concerns |
| Casanueva et al., 2016 [15] | Low | Some concerns | Low | Low | Some concerns | Low |
| Despotovic et al., 2018 [45] | Low | Some concerns | Some concerns | Low | Low | Some concerns |
| Goudarzi & Moya-Galé 2021 [46] | Low | Some concerns | Some concerns | Some concerns | Low | Some concerns |
| Joy & Umesh 2018 [58] | Low | Some concerns | Low | Low | Low | Low |
| Kadi et al., 2016 [47] | Low | Low | Some concerns | Low | Low | Low |
| Korzekwa et al., 2019 [48] | Low | Low | Low | Low | Low | Low |
| Moore et al., 2019 [37] | Some concerns | Low | Low | Low | Low | Low |
| Mulfari et al., 2022 [27] | No information | Low | Some concerns | Low | Low | Low |
| Perkoff, 2021 [38] | Low | Low | Some concerns | Low | Some concerns | Some concerns |
| Poncelet et al., 2021 [39] | Some concerns | Low | Low | Low | Some concerns | Low |
| Purohit et al., 2020 [23] | Low | High | Some concerns | Low | Low | Some concerns |
| Qi & Van Hamme 2023 [40] | Low | Some concerns | Low | Low | Low | Low |
| Renkens & Van Hamme 2018 [41] | Low | Some concerns | Low | Low | Low | Low |
| Renkens & Van Hamme 2017 [49] | Low | Low | Some concerns | Low | Low | Low |
| Sanz et al., 2022 [50] | Low | Low | Low | Low | Low | Low |
| Sarkar et al., 2022 [44] | Some concerns | Low | Low | Low | Low | Low |
| Shahamiri & Binti Salim, 2014 [28] | No information | Low | Some concerns | Low | Low | Low |
| Shahamiri & Ray 2015 [29] | Low | Low | Low | Low | Some concerns | Low |
| Thoppil et al., 2017 [30] | Some concerns | Some concerns | Low | Low | Some concerns | Some concerns |
| Tripathi et al., 2020 [31] | Low | Low | Low | Low | Low | Low |
| Tripathi et al., 2021 [32] | Low | Low | Low | Low | Low | Low |
| Wang et al., 2020 [51] | Low | Some concerns | Low | Low | Low | Low |
| Wang & Van Hamme 2023 [33] | Low | Low | Some concerns | Low | Low | Low |
| Wang et al., 2021 [34] | Low | Low | Low | Low | Low | Low |
| Wu et al., 2021 [42] | Some concerns | Low | Low | Low | Low | Low |
| Yilmaz et al., 2019 [35] | No information | Low | Some concerns | Low | Low | Some concerns |

**Multimedia Appendix 4b: Summary of Risk of Bias Assessment**

***Note***: A red circle with a minus sign indicates a high risk of bias, a yellow circle with a question mark indicates there are some concerns, and a green circle with a plus sign indicates a low risk of bias.


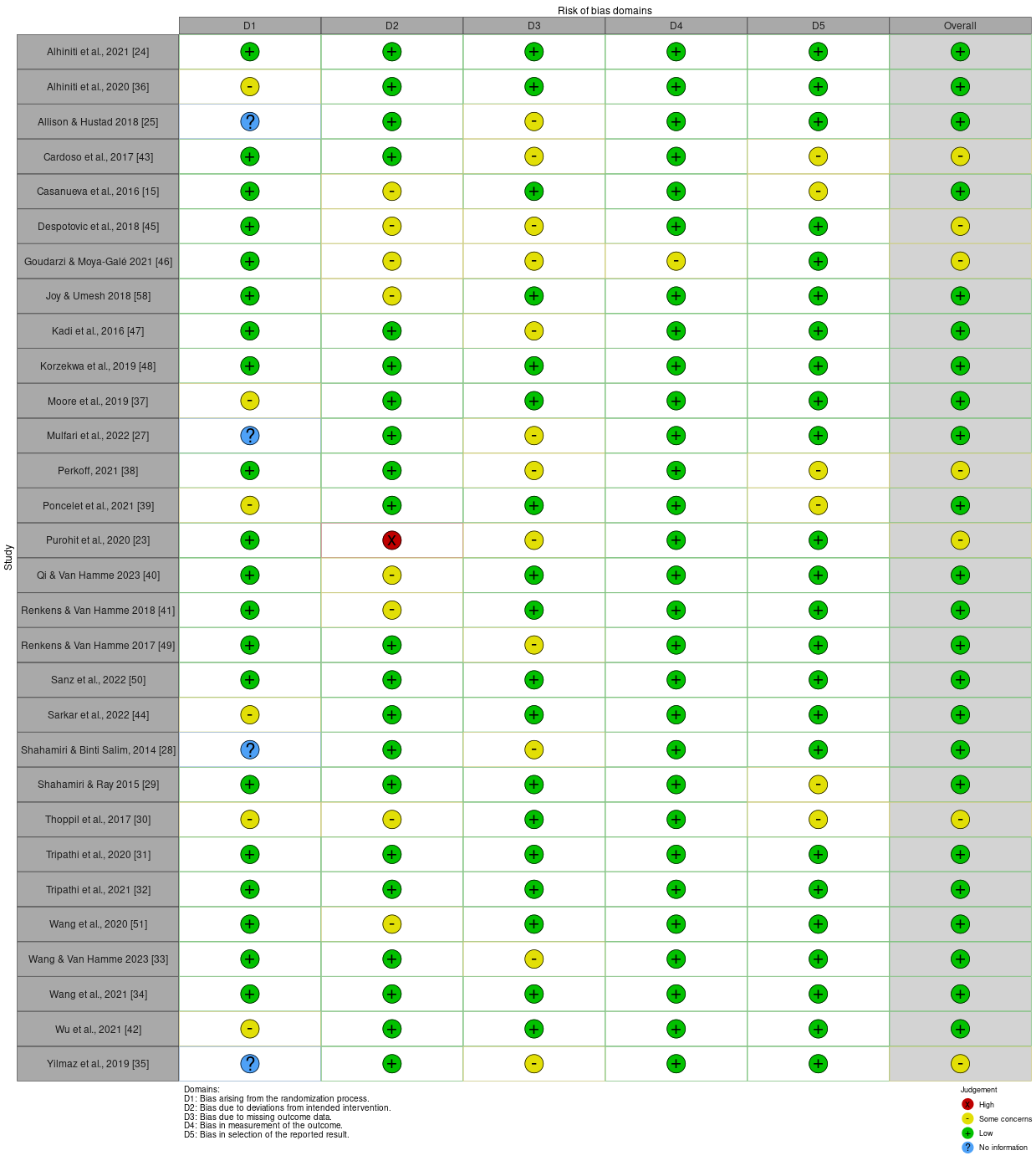

Supplement: Multimedia Appendix 4 [file rehab_v10i1e44489_app4.docx]
